# Cardiocerebrovascular risk in sensorineural hearing loss: results from the National Health and Nutrition Examination Survey 2015 to 2018

**DOI:** 10.3389/fneur.2023.1115252

**Published:** 2023-07-04

**Authors:** Jianrong Zheng, Yajing Cheng, Ying Zhan, Cong Liu, Bihua Lu, Jun Hu

**Affiliations:** ^1^Department of Neurology, Peking University Shenzhen Hospital, Shenzhen, Guangdong, China; ^2^Department of Clinical Medicine, Shantou University Medical College, Shantou, Guangdong, China

**Keywords:** sensorineural hearing loss (SNHL), cardiovascular risk, stroke, age, sex

## Abstract

**Objective:**

This study aims to determine whether the risks of cardiocerebrovascular disease are relevant to sensorineural hearing loss (SNHL) based on a national database.

**Methods:**

A total of 1,321 participants aged from 18 to 69 with complete data including medical history and audiometry from the NHANES database (2015–2018) were analyzed. All included participants had available hearing data and the average thresholds of the hearing data were measured and calculated as low-frequency pure-tone average (LFPTA; 500, 1,000, and 2,000 Hz) and high-frequency pure-tone average (HFPTA; 3,000, 4,000, 6,000, and 8,000 kHz). SNHL was defined as an average pure tone of more than or equal to 20 dB in at least one better ear. Multivariable models to assess the association between cardiocerebrovascular risks and SNHL were used in this study.

**Results:**

The prevalence of stroke was 1.6% in individuals with SNHL and 0.4% in individuals without SNHL (*p* = 0.023). A higher cardiovascular risk score was observed in SNHL patients compared to participants without SNHL (1.58 vs. 0.90, *p* < 0.001). Stroke was associated with a 3.67-fold increase in the risk of SNHL (95% CI: 1.12–12.00, *p* = 0.032) in univariable logistic regression, and the association (OR = 4.22, 95%CI = 1.28–13.93, *p* = 0.020) remained significant after adjusting for several covariates. Multivariable logistic regression models indicated a positive correlation between cardiovascular risk and SNHL (OR = 1.66, 95% CI = 1.40–1.96, *p* < 0.001), but no significant relationship was shown with all covariates adjusted. However, significant associations were found between SNHL and both age and sex in both univariable and multivariable logistic regression models.

**Conclusion:**

Our findings suggested that a higher cardiocerebrovascular risk burden was associated with an increased risk of SNHL, and the relationship may be influenced by age and sex. Future longitudinal studies are needed to investigate the mechanistic and pathologic vascular hypothesis of SNHL.

## Introduction

Hearing loss is a chronic disease that can affect people's quality of life and cause a heavy socioeconomic burden (1). It is estimated that over one in every 10 people will have disabling hearing loss requiring hearing rehabilitation (1). Sensorineural hearing loss (SNHL) is characterized by an increase in hearing thresholds of more than 20 dB in pure-tone audiometry, which results from damage of the hair cells in the cochlea, spiral ganglia, auditory nerve, or nuclei and their processing fibers in the brain (1–3). It is associated with multiple neuropsychiatric dysfunctions, such as cognitive impairment, drop attack, and depression (4–7).

Cardiovascular diseases, including ischemic heart disease and stroke, are responsible for ~22% of total deaths (1). Recent reports indicated that there were almost 19 million deaths attributed to cardiovascular diseases globally, representing an increase of 18.7% compared to 2010 (8). This global health problem is threatening the public and is probably associated with hearing loss. Previous studies have reported a relationship between a higher vascular risk profile and SNHL but have yielded different conclusions. Although the pathologic factors of SNHL vary at different periods of life in most cases, viral infections, vascular disorders, and autoimmune diseases are thought to be the most common causes (1, 9). The inner ear is especially sensitive to the blood supply, which is regulated by adrenergic receptors, plasma viscosity, and platelet function (2). Supplied by two tiny arteries at the end, the cochlea is vulnerable to damage due to several important factors, such as the small diameter of the arteries and the absence of arterial branches (10). The obstruction of blood flow of the labyrinthine artery or its origins for more than 1 min would lead to irreversible damage to audio-vestibular function (11). Microinfarcts can result from vasogenic factors, such as hypertension, diabetes, smoking, and ototoxic drugs. Ischemia and hypoxia in the inner ear may aggravate cell damage and enzyme metabolism disorder, which can further lead to a thrombosis process after reperfusion injury (12). Hence, acute or chronic vascular events, such as ischemia or infarctions, may be a potential underlying condition of SNHL (13). Comorbidities of SNHL, including myocardial infarction, hypertension, diabetes, hyperlipidemia, and stroke have been mentioned. We aimed to investigate the association between cardiovascular risk factors and SNHL using a national database and provide more evidence for this hypothesis.

## Methods

### Participants

**Figure 1:**
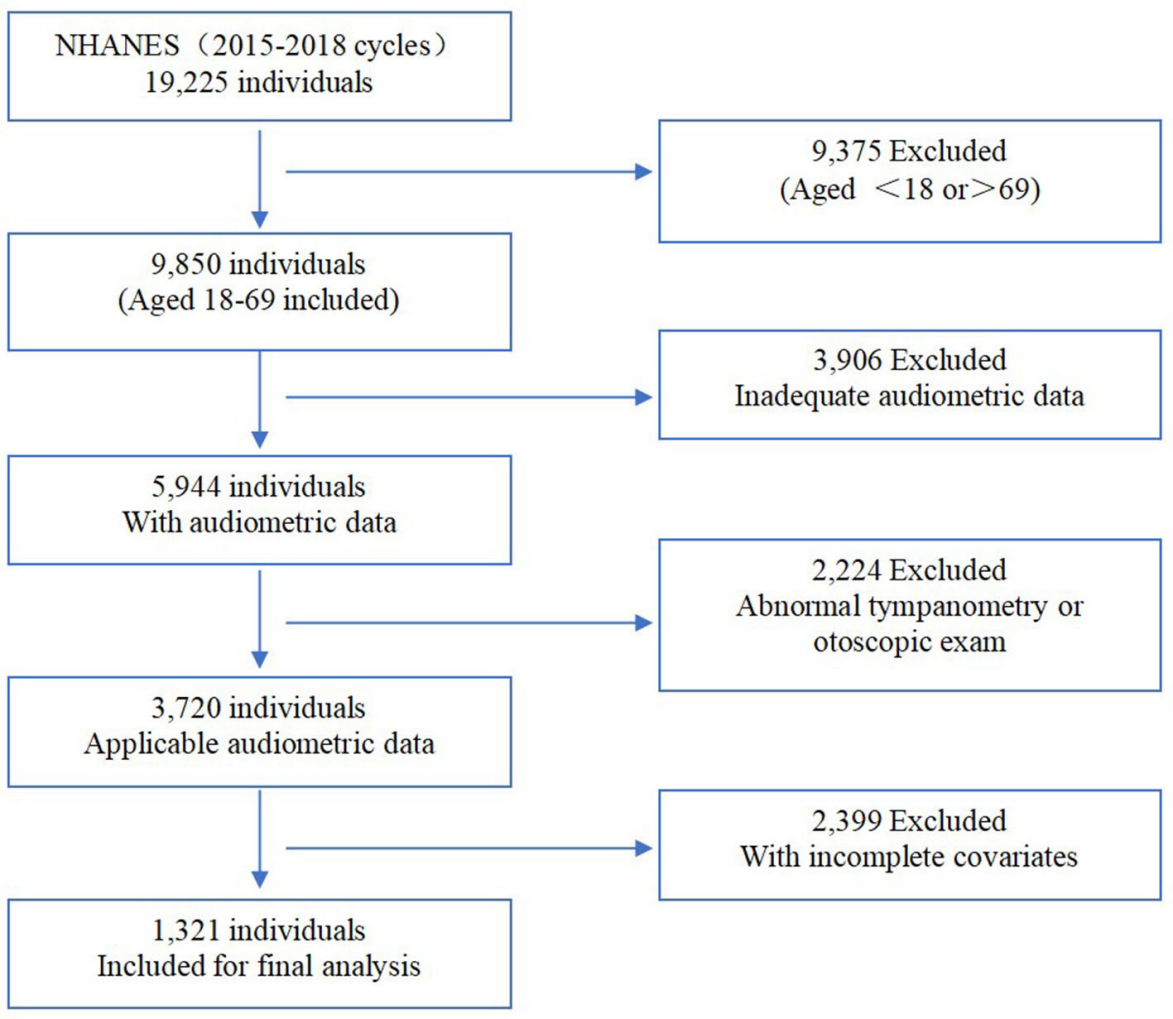
Flowchart of participant inclusion and exclusion for analysis.

The National Health and Nutrition Examination Survey (NHANES) is a representative cross-sectional health survey that registered the non-institutionalized civilian population aged 18 years and older in the United States. The survey was conducted by the National Center for Health Statistics (NCHS) of the Centers for Disease Control and Prevention and reviewed and approved by the National Center for Health Statistics Institutional Review Board. The survey was implemented every 2 years with a sample size of ~5,000 to collect information on family health and nutrition. Interviews and medical management were involved in this survey. Participants were evaluated in the NHANES interview to include their demographic information, socioeconomic situations, medical history, and health-related conditions. In addition, medical management was prepared for each assigned personnel, consisting of laboratory tests using blood and urine samples and physiological measurements, including audiometric examinations. Moreover, audiometry information was recorded with written informed consent and uploaded to the public database by professionals. Registering within two cycles in NHANES (2015–2016 and 2017–2018), there were 1,321 available participants (aged from 18 to 69) included in our research after excluding subjects with incomplete and inadequate covariates (Figure 1).

### Audiometric hearing testing

The NHANES protocol for audiometric examinations included a hearing questionnaire, otoscopic examination, tympanometry, and pure-tone air conduction audiometry. Otoscopic was performed with a Welch-Allyn otoscope (model 25020; Skaneateles Falls, NY). Tympanometry was conducted using the “Titan” Tympanometer, a new equipment that collected raw data and produced a variable number of pressure-compliance measurements. Compliance values ranged from 0.09 to 8.89 in cubic centimeters and pressure values ranged from −299 to 199 daPa. Audiometry was performed inside a sound booth using an audiometer (model AD226; Interacoustic, Assens, Denmark) with TDH-49P headphones (Telephonics Corp, Farmingdale, NY) and insert earphones (model EARTone 3A; Etymotic Research, Elk Grove Village, IL) in the mobile examination center (MEC). Air pure-tone audiometry thresholds were measured for each ear at 500, 1,000, 2,000, 3,000, 4,000, 6,000, and 8,000 Hz in deciding the hearing level. Testing at 1,000 Hz was repeated two times of the threshold across an intensity of −10 to 120 dB for both ears of each subject. If the difference between the two tests was >10 dB, it would not be accepted. Then, the results of the first 1,000 Hz test would be used in our analysis.

### Definition of sensorineural hearing loss (SNHL)

Based on the pure-tone average, normal hearing was defined as a pure tone of an average <20 dB (1). SNHL refers to bilateral a low-frequency pure-tone average (LFPTA) or high-frequency pure-tone average (HFPTA) of 20 dB or greater, or at least in the better ear, while excluding possible mixed or conductive hearing loss (3). NHANES 2015 to 2018 was composed of an audiometric questionnaire, otoscopic screening exam, tympanometry, and pure-tone air conduction audiometry to assess the participants' hearing condition. A normal finding from the otoscopic examination consisted of normal screening, no collapsing ear canals, and no other ear exam abnormalities. Participants with any problems listed above would be considered as probably suffering from conductive hearing loss and eliminated. For reference, the tympanogram type was supplemental evidence for identifying a hearing loss as conductive or sensorineural, which was further categorized into five types in NHANES. SNHL was defined as hearing loss that had type A tympanograms with a peak admittance of 0.3 ml or greater. Then, the LFPTA and HFPTA were calculated using 500, 1,000, and 2,000 Hz and 3,000, 4,000, 6,000, and 8,000 Hz, respectively.

### Covariates

Covariates included demographic information, medical history, and health-related behaviors. Demographic variables consisting of age, gender, ethnicity, poverty, and educational situations were all self-reported. The poverty-ratio index, referring to the ratio of family income to the local poverty threshold, was used to determine the family's income conditions. Drinking history was defined as alcohol consumption of any alcoholic beverages at least once a week, which was equivalent to five times a month or 53 times a year in the past year (14). A smoker was regarded as a participant who had smoked at least 100 cigarettes in his/her lifetime. According to the Compendium of Physical Activities, physical activity was evaluated by metabolic equivalent task hours for each week (MET-h/week), which was calculated by multiplying the amount of time spent in each activity with its assigned metabolic equivalent intensity and then summing up (15). The MET score was 4.0 for moderate work-related and leisure-time activity and 8.0 for vigorous activity, suggested by the NHANES guidelines. A sum of physical activity ≥3,000 MET was considered effective in our study (16). Data from the questionnaire-based interview and medical examination were used to identify participants' disease diagnoses by professional personnel who ensured the accuracy and quality. Participants who replied “Yes” to the question “Has a doctor or other health professional ever told you that you had stroke?” would be identified and registered as patients having a stroke history. Cardiovascular disease (CVD) was considered positive when interview information showed a history of coronary heart disease, congestive heart failure, heart attack, angina, or stroke. Physical examinations and laboratory tests were carried out by trained specialized personnel. Body mass index (BMI) was calculated as weight in kilograms divided by squared height in meters (kg/m^2^). Hypertension was defined as a participant who had a mean systolic blood pressure (SBP) of ≥140 mmHg, or a mean diastolic blood pressure (DBP) of ≥90 mmHg, or took antihypertensive medication. Hyperglycemia was categorized as impaired fasting glucose (IFG), impaired glucose tolerance (IGT), and diabetes mellitus. Diabetes mellitus was defined based on the participants' self-reported diagnosis or intake of antidiabetic drugs, in addition to fasting blood glucose (FBG) of ≥7.0 mmol/L (126 mg/dL) or random blood glucose (RBG) of ≥11.1 mmol/L (200 mg/dL). Participants who met at least one of the following criteria would be diagnosed as hyperlipidemia: (a) participants with confirmed diagnosed by doctors or other professionals, (b) participants with hypertriglyceridemia: TG ≥ 150 mg/dL, (c) participants with hypercholesteremia: TC ≥ 200 mg/dL (5.18 mmol/L), LDL-C ≥ 130 mg/dL (3.37 mmol/L), or HDL <40 mg/dL (1.04 mmol/L) for male, while <50 mg/dL (1.30 mmol/L) for female, and (d) participants taking lipid-lowering medication.

To avoid multicollinearity, we calculated a cardiovascular risk score (CRS) to aggregate several risk factors (7, 17). One point was assigned for each of the four present risk factors, including CVD, hypertension, hyperlipidemia, and/or history of stroke. In addition, 1 point was added for IGT and 2 points for diabetes mellitus. The score ranged from 0 to 6.

### Statistical analysis

We used the “nhanesR package” in R software (Version 0.9.3.4) for data sampling and weight calculation. Based on data from NHANES, 4-year weight was calculated to attain unbiased and representative estimates. A complex sample survey design was used in this study, and weighting calculation was utilized to communicate the prevalence of the disease. Statistical analyses were implemented using “survey Package” and SPSS (Version 22.0 IBM Corporation, Armonk, New York). Continuous variables were expressed by mean ± standard error (SE), and categorical variables were presented as percentages. *T*-tests and Pearson's chi-square test were used for the comparisons of variables between the SNHL status groups as well as between the stroke status groups. Risk factors that were probably associated with SNHL and stroke would be considered covariables in binary logistic regressions. Logistic regression with odds ratios (OR) and a 95% confidence interval (CI) adjusted for all covariables was used to examine the relationship between stroke and SNHL. Considering that there might be a multiple linear relationship in the covariables, relationships between SNHL and other variables (including age, sex, and CRS) would be measured in subsequent logistic regression models that adjusted for other variates. Statistical significance was set at *p* ≤ 0.05.

## Results

### Baseline characteristic

There is a prevalence of 46.3% (95% CI = 40.2%−52.6%) of participants with SNHL, indicating probably 16,067,973 SNHL patients in the United States. While the prevalence of stroke was found as 1.0% (95% CI = 0.6%−1.6%), suggesting overall there were 342,512 stroke patients.

The baseline characteristics of participants are listed in Tables 1, 2. Of the 1,321 participants, 595 (46.3% ± 3.0%) had SNHL, and 22 (1.0% ± 0.2%) were diagnosed with stroke. Among SNHL patients (men, 51.2%), the mean age was 51.4 ± 0.7 years. For their medical history, 40.2% of SNHL patients had hypertension, 16.6% had diabetes mellitus, and 75.8% had hyperlipidemia. Patients with a history of stroke (men, 46.5%) had a mean age of 50.0 ± 3.6 years. Of these patients, 63.0% had hypertension, 21.8% had diabetes mellitus, and 84.7% had hyperlipidemia. All of them had coronary heart disease.

**Table 1:** Clinical characteristics of all 1,321 subjects among subjects with SNHL and without SNHL.

**Variables**	**Total**	**SNHL**	**Control**	***p-*value**
*N*	1,321	595	726	
Sex (%)				0.009
Male	45.5 (1.6)	51.2 (2.9)	40.6 (2.0)	
Female	54.5 (1.6)	48.8 (2.9)	59.4 (2.0)	
Age, years	43.1 (0.8)	51.4 (0.7)	35.9 (0.6)	<0.001
BMI, kg/m^2^	29.5 (0.4)	30.4 (0.4)	28.6 (0.4)	<0.001
Race (%)				0.001
Americans	8.1 (1.7)	6.3 (1.5)	9.5 (2.1)	
Non-Hispanic White	69.6 (3.6)	75.6 (4.1)	64.5 (3.3)	
Non-Hispanic Black	8.6 (1.7)	7.1 (1.7)	9.9 (1.8)	
Other Hispanic	6.0 (1.1)	5.7 (1.2)	6.2 (1.3)	
Others	7.7 (1.3)	5.2 (1.4)	9.8 (1.4)	
Marital status (%)				<0.001
Married	58.7 (2.9)	70.4 (3.6)	48.7 (2.9)	
Widowed	2.1 (0.5)	3.5 (1.0)	0.8 (0.5)	
Divorced	7.7 (0.8)	8.2 (1.2)	7.2 (1.0)	
Separated	2.4 (0.4)	2.0 (0.5)	2.7 (0.8)	
Never married	16.4 (1.8)	6.7 (1.4)	24.8 (2.3)	
Living with partner	12.7 (1.6)	9.2 (1.7)	15.8 (2.3)	
Education level (%)				0.062
<9th grade	2.8 (0.7)	3.1 (0.7)	2.6 (0.9)	
9–11th grade (Includes 12th grade with no diploma)	6.6 (0.9)	7.0 (1.1)	6.2 (1.2)	
High school grade/GED or equivalent	18.9 (1.7)	21.9 (3.0)	16.4 (1.5)	
Some college or AA degree	31.9 (1.8)	33.0 (2.6)	31.0 (1.9)	
College graduate or above	39.8 (3.3)	35.1 (3.9)	43.8 (3.5)	
PRI, mean	3.2 (0.1)	3.3 (0.2)	3.1 (0.1)	<0.001
Hypertension (%)	29.3 (1.6)	40.2 (3.0)	19.8 (1.9)	<0.001
Diabetes mellitus (%)	10.9 (0.9)	16.6 (1.9)	6.0 (1.0)	<0.001
Hyperlipidemia (%)	63.5 (1.9)	75.8 (2.6)	52.9 (2.4)	<0.001
Coronary heart disease (%)	3.6 (0.6)	5.7 (1.3)	1.7 (0.6)	<0.001
CRS, mean	1.22 (0.04)	1.58 (0.07)	0.90 (0.05)	<0.001
Stroke (%)	1.0 (0.2)	1.6 (0.5)	0.4 (0.2)	0.023
Smoking (%)	41.6 (2.1)	49.3 (2.9)	35.0 (2.0)	<0.001
Drinking (%)	41.0 (2.6)	37.1 (3.0)	44.4 (3.0)	0.021
Physical activities (%)	45.0 (2.1)	44.2 (2.6)	45.7 (2.6)	0.637

Data are expressed as mean or percent (SE).

BMI, body mass index; PRI, poverty-ratio index; CRS, cardiovascular risk score.

**Table 2:** Clinical characteristics of all 1,321 subjects among subjects with stroke and without stroke.

**Variables**	**Total**	**Stroke**	**Non-stroke**	***p-*value**
*N*	1,321	22	1,299	
Sex (%)				0.942
Male	45.5 (1.6)	46.5 (14.4)	45.5 (1.5)	
Female	54.5 (1.6)	53.5 (14.4)	54.5 (1.5)	
Age, years	43.1 (0.8)	50.0 (3.6)	43.0 (0.8)	<0.001
BMI, kg/m^2^	29.5 (0.4)	33.2 (2.7)	29.4 (0.4)	<0.001
Race (%)				0.025
Americans	8.1 (1.7)	7.0 (5.5)	8.1 (1.7)	
Non-Hispanic White	69.6 (3.6)	44.9 (15.2)	69.9 (3.6)	
Non-Hispanic Black	8.6 (1.7)	30.1 (12.0)	8.4 (1.6)	
Other Hispanic	6.0 (1.1)	13.6 (7.7)	5.9 (1.1)	
Others	7.7 (1.3)	4.4 (4.6)	7.7 (1.3)	
Marital status (%)				0.013
Married	58.7 (2.9)	43.1 (13.5)	58.9 (3.0)	
Widowed	2.1 (0.5)	14.2 (6.9)	1.9 (0.5)	
Divorced	7.7 (0.8)	11.9 (7.5)	7.6 (0.7)	
Separated	2.4 (0.4)	3.5 (3.6)	2.4 (0.4)	
Never married	16.4 (1.8)	20.6 (9.1)	16.4 (1.9)	
Living with partner	12.7 (1.6)	6.5 (6.6)	12.8 (1.6)	
Education level (%)				0.004
<9th grade	2.8 (0.7)	0.0 (0.0)	2.9 (0.7)	
9–11th grade (Includes 12th grade with no diploma)	6.6 (0.9)	13.0 (8.1)	6.5 (0.9)	
High school grade/GED or equivalent	18.9 (1.7)	58.0 (14.6)	18.5 (1.7)	
Some college or AA degree	31.9 (1.8)	20.0 (7.8)	32.1 (1.8)	
College graduate or above	39.8 (3.3)	9.1 (6.0)	40.1 (3.3)	
PRI, mean	3.2 (0.1)	1.4 (0.2)	3.2 (0.1)	<0.001
Hypertension (%)	29.3 (1.6)	63.0 (14.4)	28.9 (1.6)	0.018
Diabetes mellitus (%)	10.9 (0.9)	21.8 (9.8)	10.8 (0.9)	0.162
Hyperlipidemia (%)	63.5 (1.9)	84.7 (7.8)	63.3 (1.9)	0.047
Coronary heart disease (%)	3.6 (0.6)	100.0 (0.0)	2.6 (0.5)	<0.001
CRS, mean	1.22 (0.04)	3.91 (0.20)	1.19 (0.04)	<0.001
SNHL (%)	46.3 (3.0)	75.8 (10.8)	46.0 (3.0)	0.023
Smoking (%)	41.6 (2.1)	45.9 (13.9)	41.6 (2.1)	0.761
Drinking (%)	41.0 (2.6)	21.1 (9.5)	41.2 (2.6)	0.085
Physical activities (%)	45.0 (2.1)	51.1 (14.8)	44.9 (2.2)	0.695

Data are expressed as mean or percent (SE).

BMI, body mass index; PRI, poverty-ratio index; CRS, cardiovascular risk score; SNHL, sensorineural hearing loss.

### Demographic and cardiovascular indicators between groups

**Figure 2:**
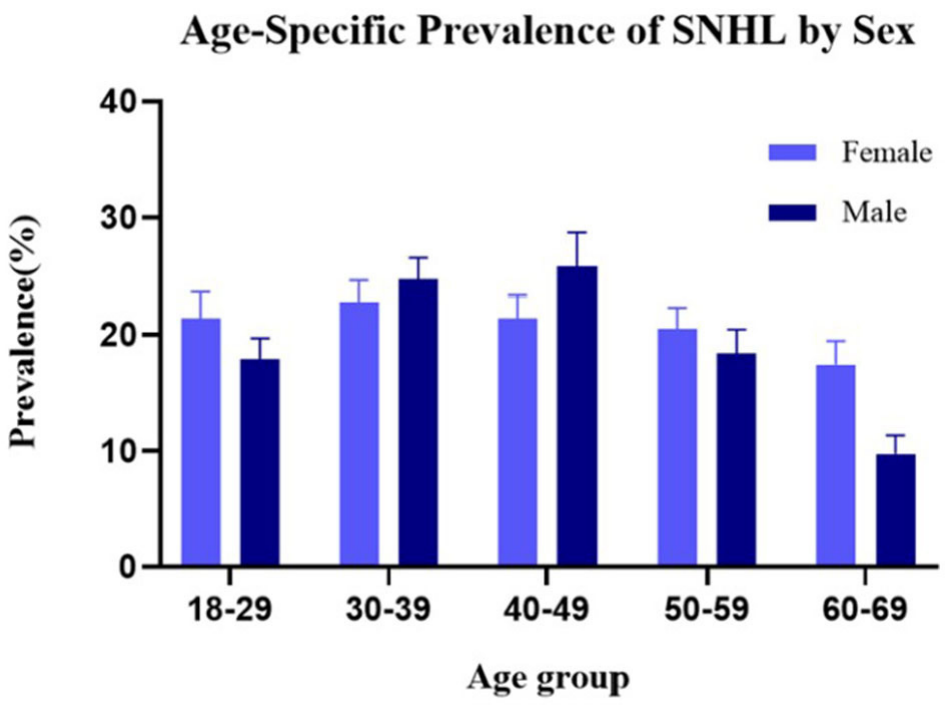
Prevalence of SNHL based on the classification of age and sex.

Participants were classified into the SNHL group and control group (people without SNHL). Table 1 shows the characteristics of the two groups and their differences. The weight prevalence of SNHL in men (*p* = 0.009), elders (*p* < 0.001), stroke (*p* = 0.023), hypertension (*p* < 0.001), diabetes mellitus (*p* < 0.001), hyperlipidemia (*p* < 0.001), coronary heart disease (*p* < 0.001), smoking (*p* < 0.001), and alcohol consumption (*p* = 0.021) was significantly higher compared with that of the control group. Statistically significant differences were found between the two groups in race and marital status. Furthermore, a higher cardiovascular risk score was observed in SNHL patients compared with healthy hearing participants (1.58 vs. 0.90, *p* < 0.001). No significant difference was found in other baseline data. Sex distribution based on the age-specific sections is shown in Figure 2. The weight proportion of SNHL in men was higher than that in women in the younger age group (21.4 vs. 17.9%, aged 18–29) and in the older age group (21.4 vs. 25.8%, aged 50–59; 17.4 vs. 9.7%, aged 60–69), but was lower than that in women (24.7 vs. 22.8%, aged 30–39; 25.8 vs. 21.4%, aged 40–49) in the middle age group. The weight proportion of SNHL varied between genders as age increased. Participants were classified into the stroke group and the non-stroke group based on their medical history. Table 2 shows the characteristics of the two groups and their differences. Among stroke patients, there was a higher weight proportion of cardiovascular diseases such as hypertension (*p* = 0.018), hyperlipidemia (*p* = 0.047), and coronary heart disease (*p* < 0.001). Similarly, the weight proportion of SNHL in the stroke group was higher (*p* = 0.023). The cardiovascular risk score was significantly higher in the stroke group compared with that in the non-stroke group (3.91 vs. 1.19, *p* < 0.001). Meanwhile, a significant difference was found in ethnicity, marital status, and educational background between the stroke group and the non-stroke group. There were no significant relationships between stroke and other factors, including sex, diabetes mellitus, smoking, alcohol use, and physical activity.

### Logistic regression models

#### Stroke and SNHL

Multivariable logistic regression models were used to assess the effect of stroke on SNHL status (Table 3). Compared with individuals without stroke, individuals with stroke were more likely to have SNHL (OR = 3.67, 95% CI = 1.12–12.00, *p* = 0.032). When controlling for ethnicity, a significant correlation still existed (OR = 4.17, 95% CI = 1.31–13.21, *p* = 0.017). Further controlling for ethnicity and sex suggested that individuals with stroke were more likely to have SNHL (OR = 4.22, 95% CI = 1.28–13.93, *p* = 0.020). Individuals with stroke still had a slightly higher risk of SNHL (OR = 3.74, 95% CI = 0.93–16.95, *p* = 0.063) when additionally controlling for marital status, educational background, and familial economic status. There was no significant relationship (OR = 2.21, 95% CI = 0.20–23.90, *p* = 0.501) between stroke and SNHL after the fully adjusted logistic regression that included other covariables, such as BMI, hypertension, diabetes mellitus, hyperlipidemia, coronary heart disease, smoking, alcohol use, and physical activity.

**Table 3:** Association between stroke and SNHL.

	**OR**	**95%CI**	***p-*value**
Unadjusted model	3.67	1.12–12.00	0.032
Model 1	4.17	1.31–13.21	0.017
Model 2	4.22	1.28–13.93	0.020
Model 3	3.74	0.93–16.95	0.063
Model 4	2.21	0.20–23.90	0.501

Model 1, adjusted for race.

Model 2, further adjusted for sex.

Model 3, further adjusted for education, marriage, and PRI, and

Model 4, further adjusted for age, BMI, hypertension, diabetes mellitus, hyperlipidemia, coronary heart disease, smoking, drinking, and physical activity.

#### Cardiovascular risk score (CRS) and SNHL

Other multivariable logistic regression models that described the influence of CRS are illustrated in Table 4. Higher CRS may contribute to a higher risk of SNHL according to unadjusted logistic regression (OR = 1.73, 95% CI = 1.49–2.00, *p* < 0.001). After controlling for sex, ethnicity, education, and income, CRS still indicated an influence on the risk of sensorineural hearing loss (OR=1.66, 95% CI = 1.40–1.96, *p* < 0.001). No significance was indicated after the fully adjusted logistic regression that additionally included other covariates (OR = 1.07, 95% CI = 0.88–1.31, *p* = 0.110).

**Table 4:** Association between the cardiovascular risk score and SNHL.

	**OR**	**95%CI**	***p-*value**
Unadjusted model	1.73	1.49–2.00	<0.001
Model 1	1.77	1.53–2.05	<0.001
Model 2	1.79	1.53–2.08	<0.001
Model 3	1.66	1.40–1.96	<0.001
Model 4	1.07	0.88–1.31	0.110

Model 1, adjusted for race.

Model 2, adjusted for sex.

Model 3, adjusted for education, marriage, and PRI, and

Model 4, adjusted for age, BMI, smoking, drinking, and physical activity.

#### Age, sex, and SNHL

In unadjusted multivariable logistic regression models (Table 5), elders and men were more likely to have SNHL (Age: OR = 1.12, 95% CI = 1.10–1.14, *p* < 0.001; Sex: OR = 1.54, 95% CI = 1.12–2.10, *p* = 0.009). The correlation remained statistically significant when adjusting for all the other covariates (Age: OR = 1.12, 95% CI = 1.11–1.14, *p* < 0.001; Sex: OR = 1.89, 95% CI = 1.28–2.77, *p* = 0.002).

**Table 5:** Association between age (or) sex and SNHL.

	**Unadjusted model**	**Adjusted model**
**Age**		
OR	1.12	1.12
95%CI	1.10–1.14	1.11–1.14
*p-*value	<0.001	<0.001
**Sex (male)**		
OR	1.54	1.89
95%CI	1.12–2.10	1.28–2.77
*p-*value	0.009	0.002

Adjusted model: adjusted for race, education, marriage, PRI, BMI, hypertension, diabetes mellitus, hyperlipidemia, coronary heart disease, stroke, smoking, alcohol, and physical activity.

### Diagnostic models in the ROC curve

**Figure 3:**
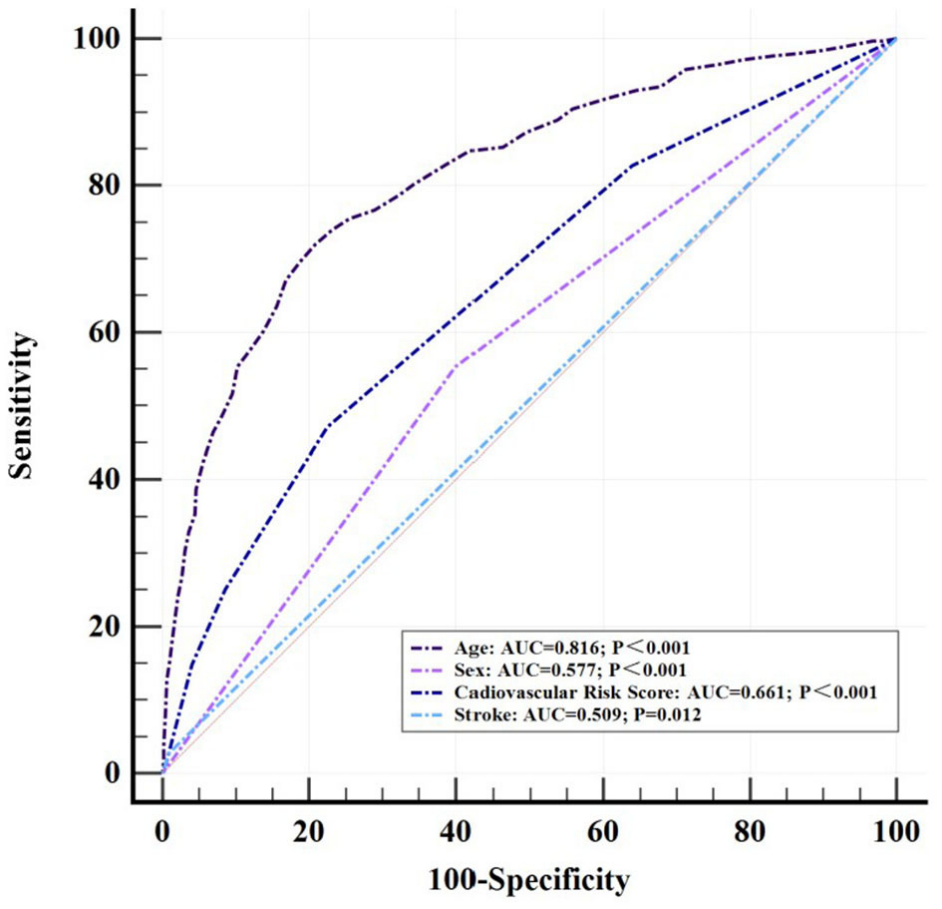
The ROC curve.

Logistic regression analysis was performed to examine the variables that were significantly different between the SNHL group and the control group. Age (OR = 1.12, 95% CI = 1.10–1.14, *p* < 0.001), sex (OR = 1.54, 95% CI = 1.12–2.10, *p* = 0.009), cardiovascular risk score (OR = 1.73, 95% CI = 1.49–2.00, *p* < 0.001), and stroke (OR = 3.67, 95% CI = 1.12–12.00, *p* = 0.03) were included, respectively, in the construction of a diagnostic model for SNHL with receiver-operating characteristics (ROC) (Figure 3). The AUC of the diagnostic model based on the age threshold was the highest at 0.816 (95% CI = 0.794–0.836, *p* < 0.001) with a sensitivity of 71.93% and a specificity of 79.20%. Though a relatively satisfied AUC of 0.661 (95% CI = 0.634–0.686, *p* < 0.001) was identified in the diagnostic model based on the CRS threshold, the AUC of 0.509 (95% CI = 0.482–0.537, *p* = 0.012) with a high specificity of 99.17% was of low accuracy based on stroke threshold.

### Sensitivity analysis

Sensorineural hearing loss was classified into two groups based on the pure-tone average of an LFPTA or HFPTA more than or equal to 20 dB in at least the better ear. Similarly, higher CRS is associated with a higher risk of low-frequency sensorineural hearing loss (LSNHL) (OR = 1.42, 95% CI = 1.12–1.81, *p* = 0.006 in Supplementary Table 1) and high-frequency sensorineural hearing loss (HSNHL) (OR = 1.58, 95% CI = 1.39–1.80, *p* < 0.001 in Supplementary Table 1), respectively, in the univariate analysis. However, no significant correlations were found after controlling the confounders. Stroke was associated with high-frequency hearing loss (OR = 5.25, 95% CI = 1.73–16.01, *p* = 0.005 in Supplementary Table 1) but not low-frequency hearing loss (OR = 2.80, 95% CI = 0.86–9.19, *p* = 0.086 in Supplementary Table 1). No significant association was found between stroke and LSNHL or HSNHL, after adjusting for all confounders.

In a second sensitivity analysis in Supplementary Table 2, the subgroup was restricted to individuals aged ≥50 years. Our findings did not show statistical significance in univariate analysis. There might be an increased risk of sensorineural hearing loss in post-stroke patients (OR = 2.63, 95% CI = 0.46–15.05, *p* = 0.267) or in patients with higher cardiovascular risk (OR = 1.22, 95% CI = 0.92–1.62, *p* = 0.152).

## Discussion

A positive association between cardiocerebrovascular risk and sensorineural hearing loss (SNHL) was observed among the population classified by hearing level in our study. The absence of independent associations between cardiovascular risk and SNHL may be due to the covariates that were independently associated with hearing loss, such as age and sex. In addition, the diagnostic models constructed by age, sex, CRS, and stroke were illustrated with a low-to-moderate accuracy, in which age and sex were superior to the others. More effective factors should be figured out in a comprehensive predictive model in the following research.

Accounting for the majority of all types of hearing loss, SNHL refers to any causes of hearing loss resulting from damage in the cochlea, auditory nerve, or central nervous system (18). Pathogenesis of hearing loss involves injured hair cells, cochlear neuron deficits, atria vascularis, a combined injury, or aging itself (19–21). Early in 1987, *Böhme* demonstrated that arterial sclerotic vascular disease should be considered as one of the multifactorial geneses of hearing disorders in older adults (22). The sudden onset of SNHL is thought to be caused by an interruption of the vascular supply to the cochlea in the inner ear (18, 23, 24). As the terminal branch of the anterior inferior cerebellar artery (AICA), the internal auditory artery (IAA) irrigates the cochlea and the vestibular labyrinth. Occlusion of the IAA is mostly due to thrombotic narrowing of the AICA itself or the orifice of AICA at the basilar artery, which may cause a sudden auditory disorder, sometimes combined with a loss of vestibular function such as vertigo (25–27). Audio-vestibular loss is regarded as an important sign for the diagnosis of AICA infarction (26–28). The process of hearing depends on the hair cell converting the mechanical energy of sound waves into electrical signals that are transmitted through the nerve fibers. The process is reliant on adequate blood flow to the inner ear (29). Adrenergic receptors, plasma viscosity, platelet function, and vascular integrity play a key role in maintaining a normal blood supply (8, 30). Any vascular dysfunctions that result in ischemia, minor infarction, and microbleed may impair the cochlear perfusion in the same way that coronary or cerebral infarction occurs (8). Therefore, it might be claimed that SNHL is a marker for stroke incidence or previous stroke rather than being a risk factor for it. The findings mentioned above strengthen the hypothesis of the vascular origin of SNHL. Vascular involvement in the inner ear leading to SNHL acquires more attention (31, 32).

People who possess higher cardiocerebrovascular risk may suffer from a higher risk of SNHL. A remarkably higher risk of a sudden SNHL in stroke patients was found compared with that in non-stroke patients within a 5-year follow-up after their stroke onset (33). This prospective link may result from the overlapping of cardiovascular risk factors. The prevalence of hypertension, diabetes mellitus, hyperlipemia, and coronary heart disease in both the SNHL group and the stroke group is higher than that of their control groups. As a result, our findings may contribute to the hypothesis. Hyperglycemia, hypertension, and hypercholesterolemia are risk factors for endothelial dysfunction, oxidative stress, and vascular inflammation, which may represent common inner ear pathology associated with vascular injury (34, 35). There are risk factors associated with atherosclerotic disease and hearing loss, including aging (34, 36). The association between vascular risk and SNHL may be weakened when adjusting for age, which is similar to our results. Thus, aging increases the risk of hearing loss directly and indirectly (34). Sex differences are also present in the auditory system (37). Men are more likely to suffer from hearing loss (38, 39) while women probably benefit from estrogen and its related signaling pathway to avoid the damage from atherosclerotic disease and hearing loss (40). Other vascular risk factors may be positively associated with SNHL as well. For example, smoking (23, 41), alcohol consumption, physical activity, and obesity seem to increase the risk of SNHL. However, it is inconsistent with the findings in our study (23, 42, 43).

Moreover, previous research indicated that SNHL may predict the risk of stroke. Based on the population from Korea and Taiwan, three studies investigated and found that sudden SNHL resulted in a significantly increased risk of the occurrence of stroke (44–46). Two Chinese longitudinal studies based on the Dongfeng-Tongji Cohort indicated a similarly positive result, showing that the risk of stroke may be related to an increased level of hearing loss (15, 47). However, conflicting results were also found in other cohort studies. With an adequate sample size, Ciorba et al. identified a lower risk of stroke in people with hearing loss (19), leaving confusion on the pathogenesis. A meta-analysis performed by *Lammers* et al. indicated the relationship by demonstrating a pooled odds ratio of stroke as 1.42 in idiopathic sudden SNHL patients (95% CI = 1.15–1.75) and *I*^2^ as 55.0% (*p* = 0.001) (48). An increased risk of stroke with a pooled HR of 1.44 (95% CI = 1.15–1.74) and *I*^2^ of 55.0% (*p* = 0.038) in SNHL was indicated by *Khosravipour* et al. (8). However, the calculated data in these two analyses were similar and of moderate quality of evidence partly because they included some of the same studies. Hence, direct evidence supporting the correlation between stroke and SNHL is still limited, which requires investigation into the effects of additional vascular confounding factors on the relationship. In our study, age and sex were identified as independent risk factors for SNHL. These factors play vital roles in cardiocerebrovascular diseases, including stroke, and act as intermediate factors contributing to the results. This indicates a correlation between SNHL and cardiocerebrovascular disease.

There are several limitations in our present study and analysis. First, the comprehensive causal relationship between cardiocerebrovascular risk and SNHL cannot be fully addressed in this cross-sectional study. Second, sample selection is limited because of the partial information from the NHANES dataset. Since the NHANES did not include individuals who were institutionalized, the generalizability of our findings may be underestimated. Compared to previous larger cohorts and meta-analysis, the sample size was relatively small. In addition, the diagnosis of sensorineural hearing loss in our study mainly depended on pure-tone audiometry with normal otoscopic examination and tympanograms with peaks >0.3 ml, which are probably biased toward the conductive component in hearing loss. Third, although we adjusted for several major confounders, there may still be other potential factors that could confound the relationship between cardiocerebrovascular risk and SNHL. For example, metabolic syndrome (49), renal failure (50), and thromboembolic events can also be involved.

## Conclusion

This present cross-sectional study based on the NHANES population database established that cardiocerebrovascular risk factors are associated with sensorineural hearing loss. However, aging and sex may be the more direct factors underlying this relationship. Our findings may enrich the understanding of the pathology of SNHL and highlight the importance of early caution and intervention in vascular events to prevent hearing loss. Further confirmation through comparative longitudinal prospective studies is needed in the future.

## Data availability statement

The original contributions presented in the study are included in the article/Supplementary material, further inquiries can be directed to the corresponding author.

## Ethics statement

Ethical review and approval was not required for the study on human participants in accordance with the local legislation and institutional requirements. Written informed consent was not provided because data are available in a public, open access repository.

## Author contributions

Study concept and design was fulfilled by JZ and JH. JZ wrote the manuscript which was modified by JH. JZ, CL, and BL performed the statistical analysis which was collated by YC and YZ. All authors have contributed significantly to the final version of the manuscript.
