# Supplementary material for: Cardiocerebrovascular risk in sensorineural hearing loss: results from the National Health and Nutrition Examination Survey 2015 to 2018
Source: Front Neurol. 2023 Jul 4;14:1115252. doi: 10.3389/fneur.2023.1115252 (PMC10353435; doi:10.3389/fneur.2023.1115252)
Supplement: Supplementary file 1 [file Data_Sheet_1.docx]

Supplementary Material

Table S1 The association between stroke (or) cardiovascular risk score (CRS) and LFPTA

|  | Unadjusted model | Model 1 | Model 2 | Model 3 |
| --- | --- | --- | --- | --- |
| Stroke |  |  |  |  |
| OR | 2.80 | 2.79 | 2.74 | 3.17 |
| 95%CI | 0.86-9.19 | 0.82-9.52 | 0.42-17.83 | 0.40-25.54 |
| *p-*value | 0.086 | 0.099 | 0.281 | 0.267 |
| CRS |  |  |  |  |
| OR | 1.42 | 1.43 | 0.60 | 0.78 |
| 95%CI | 1.12-1.81 | 1.11-1.83 | 0.20-1.77 | 0.22-2.81 |
| *p-*value | 0.006 | 0.007 | 0.338 | 0.698 |

Model 1: adjusted for race,

Model 2: further adjusted for BMI, hypertension, diabetes mellitus, hyperlipidemia, coronary heart disease, stroke, smoking, alcohol and physical activity,

Model 3: further adjusted for sex and age.

The association between stroke (or) cardiovascular risk score (CRS) and HFPTA

|  | Unadjusted model | Model 1 | Model 2 | Model 3 |
| --- | --- | --- | --- | --- |
| Stroke |  |  |  |  |
| OR | 5.25 | 6.63 | 1.95 | 2.72 |
| 95%CI | 1.73-16.01 | 2.19-20.00 | 0.37-10.43 | 0.32-22.88 |
| *p-*value | 0.005 | 0.002 | 0.421 | 0.344 |
| CRS |  |  |  |  |
| OR | 1.58 | 1.63 | 0.81 | 1.07 |
| 95%CI | 1.39-1.80 | 1.43-1.86 | 0.37-1.77 | 0.36-3.18 |
| *p-*value | ＜0.001 | ＜0.001 | 0.584 | 0.901 |

Model 1: adjusted for race,

Model 2: further adjusted for BMI, hypertension, diabetes mellitus, hyperlipidemia, coronary heart disease, stroke, smoking, alcohol and physical activity,

Model 3: further adjusted for sex and age.

Table S2 The association between stroke (or) cardiovascular risk score (CRS) and SNHL in people aged≥50

|  | OR | 95%CI | *p-*value |
| --- | --- | --- | --- |
| Stroke  CRS | 2.63  1.22 | 0.46-15.05  0.92-1.62 | 0.267  0.152 |
